# Crystal structure of di-μ-chlorido-bis­{chlorido­[(−)-5,6-pinenebi­pyridine]­cobalt(II)} aqua­dichlorido[(−)-5,6-pinenebi­pyridine]cobalt(II)

**DOI:** 10.1107/S2056989022003589

**Published:** 2022-04-05

**Authors:** Massimo Varisco, Aurelien Crochet, Olimpia Mamula Steiner

**Affiliations:** a University of Applied Sciences of Western Switzerland, HES-SO, HEIA-FR, Boulevard de Pérolles 80, CH-1700 Fribourg, Switzerland; b Université de Fribourg, Département de Chimie, Chemin du Musée 9, CH-1700 Fribourg, Switzerland

**Keywords:** crystal structure, cobalt(II) complex, (−)-5,6-pinenebi­pyridine, hydrogen bonding

## Abstract

The crystal structure of [Co_2_Cl_4_(C_17_H_18_N_2_)_2_][CoCl_2_(C_17_H_18_N_2_)(H_2_O)] or [Co(*L*)Cl(μ-Cl)]_2_[Co(*L*)(Cl)_2_(OH_2_)], where *L* is the enanti­opure bidentate ligand (−)-5,6-pinenebi­pyridine (C_17_H_18_N_2_), has been determined. Crystals suitable for X-ray structure analysis were obtained by slow evaporation of an ethano­lic solution containing equimolar amounts of *L* and CoCl_2_·6H_2_O. The Co^II^ cations all have a coordination number of five, and in each case the coordination polyhedron is a trigonal bipyramid. The Co—N bonds lengths range from 2.037 (7) to 2.195 (7) Å, and Co—Cl bonds lengths range from 2.284 (2) to 2.509 (2) Å. The asymmetric unit contains two discrete complexes, one dinuclear and the other mononuclear. Between the two mol­ecules, two types of inter­molecular inter­actions have been evidenced: π–π stackings involving the bi­pyridine units, and O—H⋯Cl hydrogen bonds between the hydrogen atoms of the aqua ligand coordinating to the mononuclear complex and the non-bridging chlorido ligand coordinating to the dinuclear mol­ecule. These inter­actions lead to a two-dimensional supra­molecular arrangement parallel to the *ab* plane.

## Abstract 2

The crystal structure of the title compound comprises two different Co^II^ complexes, one mononuclear and the other dinuclear, with the enanti­opure, bidentate (−)-5,6-pinenebi­pyridine ligand. All three coordination polyhedra around the Co^II^ cations are distorted trigonal bipyramids.

## 1. Chemical context

Single-mol­ecule magnets (SMMs) are metal–organic compounds that are superparamagnetic below a blocking temperature. It is important to note that this type of magnetism has a mol­ecular origin, instead of the more traditional bulk-originated magnetism (Zhu *et al.*, 2013 ▸). Below the blocking temperature, a SMM exhibits magnetic hysteresis. In order to obtain a coordination compound behaving as an SMM, a paramagnetic metal cation has to be used, for example Co^II^ (Lang *et al.*, 2019 ▸). Moreover, the use of chiral ligands for these paramagnetic metal cations can lead to predetermination of their chirality and thus to the synthesis of magnetochiral materials (Liu *et al.*, 2018 ▸). The enanti­omers of 5,6-pinene bi­pyridine (C_17_H_18_N_2;_ *L*) and their derivatives have the ability to predetermine the chirality of *d* and *f* metal cations (Lama *et al.*, 2008 ▸; Mamula & von Zelewsky, 2003 ▸). 
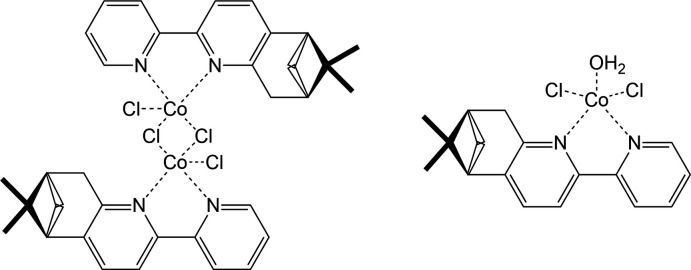


Within a current project we are investigating the metal complexes obtained with paramagnetic metal cations, *i.e.* Co^II^, and report here the crystal structure of [Co(*L*)Cl(μ-Cl)]_2_[Co(*L*)(Cl)_2_(OH_2_)] (**1**)_._

## 2. Structural commentary

**Figure 1:**
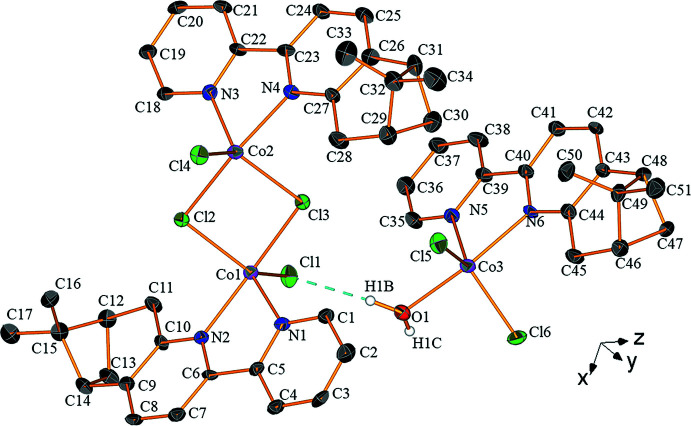
The mol­ecular structures of the two complexes present in (**1**), with the O—H⋯Cl hydrogen bond shown as a dashed line. Displacement ellipsoids are set at the 30% probability level. Carbon-bound hydrogen atoms are omitted for clarity.

The asymmetric unit of (**1**) comprises two discrete complexes (Fig. 1 ▸). The dinuclear complex possess two bidentate terminal (−)-5,6-pinenebi­pyridine ligands coordinated by two distinct Co^II^ cations (Co1, Co2) *via* their nitro­gen atoms. The two Co^II^ cations are linked by two bridging chlorido ligands (Cl2, Cl3). Each coordination sphere is completed by two additional terminal chlorido ligands (Cl1, Cl4), leading to a coordination number of 5 in each case. The mononuclear complex (Co3) also features a Co^II^ cation with a coordination number of 5. In this case, one bidentate (−)-5,6-pinenebi­pyridine, two terminal chlorido ligands (Cl5; Cl6) and an aqua ligand bind to the Co^II^ cation. The two types of complexes inter­act *via* an O—H⋯Cl hydrogen bond (indicated with a dashed line in Fig. 1 ▸; Table 1 ▸) between one hydrogen atom belonging to the aqua ligand of the mononuclear complex and a terminal chlorido ligand belonging to the dinuclear complex. The other hydrogen atom of the water mol­ecule forms another hydrogen bond with a dinuclear complex belonging to a neighbouring mol­ecule (*vide infra*).

**Figure 2:**
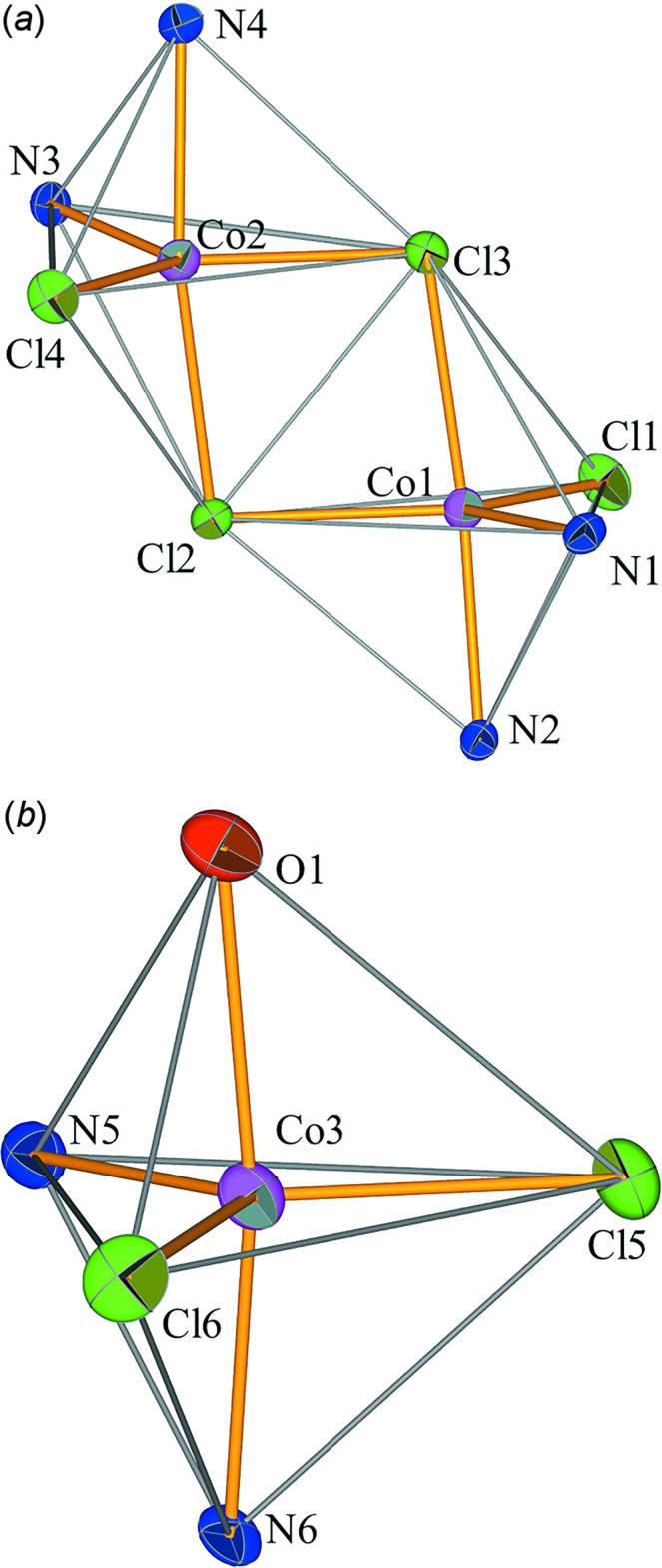
The trigonal–bipyramidal coordination spheres of the Co^II^ cations in (*a*) the dinuclear complex and (*b*) the mononuclear complex. Non-coordinating atoms are omitted for clarity.

The geometric parameters for the trigonal–bipyramidal coordination environments are similar for the three Co^II^ cations. In order to compare their coordination polyhedra, the values for the parameter τ were calculated. For a perfect trigonal–bipyramidal arrangement τ is 1, and for a perfect square-pyramidal arrangement τ is 0 (Addison *et al.*, 1984 ▸). The polyhedron around the cation in the mononuclear complex (Co3 in Fig. 2 ▸) is the closest to trigonal–bipyramidal (τ = 0.78). However, those of the cations of the dinuclear complex are not so different (τ = 0.69 for Co1, τ = 0.64 for Co2, see Fig. 2 ▸).

The Co—N bond lengths are between 2.037 (7) and 2.195 (7) Å, the Co—Cl bonds lengths are between 2.284 (2) and 2.509 (2) Å and the Co—O bond length is 2.160 (6) Å, which are all within the expected ranges (Bernhardt & Lawrance, 2003 ▸).

## 3. Supra­molecular features

**Figure 3:**
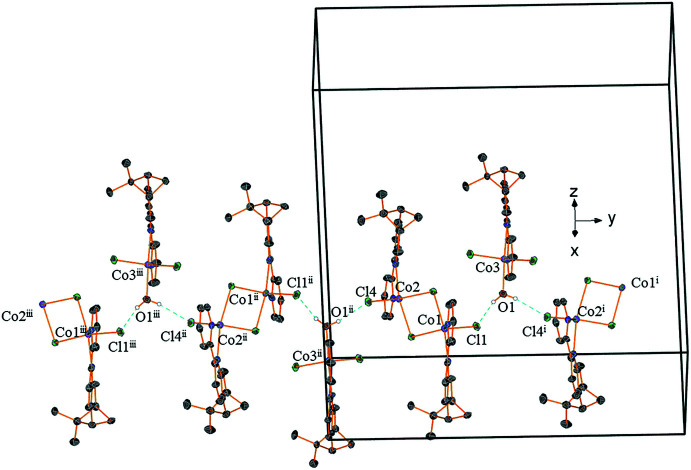
Hydrogen bonds (blue dotted lines) forming an infinite supra­molecular chain. Carbon-bound hydrogen atoms are omitted for clarity. [Symmetry codes: (i) 1 − *x*, 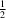 + *y*, 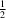 − *z*; (ii) 1 − *x*, − 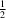 + *y*, 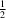 − *z*; (iii) *x*, −1 + *y*, *z*.]

In the crystal, hydrogen-bonding inter­actions occur between the dinuclear and mononuclear complexes, leading to a supra­molecular zigzag chain extending parallel to the *b* axis (Fig. 3 ▸). The hydrogen atoms of the aqua ligand of the mononuclear complex form hydrogen bonds with the terminal chlorido ligands belonging to the dinuclear complex. The bond lengths and angles (Table 1 ▸), are in the expected ranges for this type of inter­action (Steiner, 2002 ▸).

**Figure 4:**
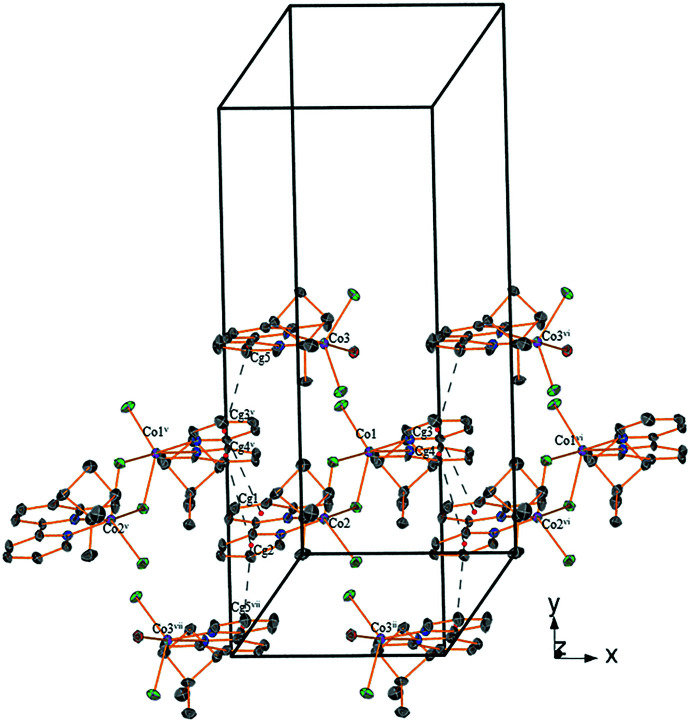
π–π stacking inter­actions shown as dotted black lines. [Symmetry codes: (ii) 1 − *x*, − 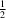 + *y*, 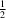 − *z*; (v) −1 + *x*, *y*, *z*; (vi) 1 + *x*, *y*, *z;* (vii) −*x*, − 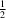 + *y*, 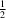 − *z*.]

This arrangement is stabilized by π–π stacking inter­actions, which are responsible for the cohesion of the structure by forming layers of alternating dinuclear and mononuclear complexes extending parallel to the *ab* plane (Fig. 4 ▸). Neighbouring dinuclear complexes are connected *via* π–π inter­actions between the bi­pyridine units whereby two π–π inter­actions are established between the two pyridine rings annelated to the pinene moiety and the two ‘free pyridines’ (the pinene-free pyridine rings of the pinene-bi­pyridine ligands). The distances between the aromatic centroids are 3.793 (5) Å (slippage 0.987 Å) and 3.940 (5) Å (slippage 1.278 Å). The two pinene bi­pyridine ligands belonging to neighbouring dinuclear complexes are connected *via* their ‘free’ pyridine entity to the ‘free’ pyridine entities of the pinenebi­pyridine ligands of the mononuclear complexes. The distances [3.625 (5) Å with a slippage of 1.137 Å, and 3.718 (5) Å with a slippage of 1.503 Å] are typical for these kinds of inter­actions (Robin & Fromm, 2006 ▸).

**Figure 5:**
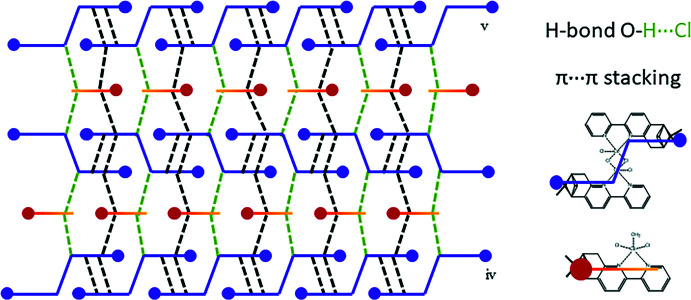
Schematic representation of the two-dimensional arrangement in the crystal structure of (**1**). [Symmetry codes: (iv) −*x*, 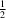 + *y*, 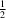 − *z*; (v) −1 + *x*, *y*, *z*.]

Considering all the inter­molecular inter­actions (hydrogen bonds and π–π stackings), the two-dimensional supra­molecular arrangement can be drawn schematically as shown in Fig. 5 ▸.

## 4. Database survey

A survey of the Cambridge Structural Database (Version 5.42, September 2021; Groom *et al.*, 2016 ▸) revealed no cobalt complexes containing the ligand (−) or (+)-5,6-pinenebi­pyridine (nor 4,5-pinenebi­pyridine). However, a few mononuclear complexes with ligands containing the 5,6-pinenebi­pyridine moiety in their skeleton have been reported. A tetra­hedral Co^II^ complex, UCUFAZ, containing a bidentate bi­pyridine ligand analogue to the ligand *L* but containing two pinene groups, has been characterized (Lötscher *et al.*, 2001 ▸). Two tridentate ligands, UKITOX and UKIVAL (Suhr *et al.*, 2002 ▸), composed of 2,2′:6′,2′′ terpyridine containing two pinene groups annelated to the terminal pyridine rings, coord­inated by a Co^II^ cation together with two chloride anions to form a complex whose geometry is pseudo-trigonal–bipyramidal. Finally, Yeung *et al.* (2009 ▸) used terpyridine ligands from the same family as the ones of Suhr *et al.* and obtained similar structures (XUDHOU and XUDJEM).

## 5. Synthesis and crystallization

A pink solution of CoCl_2_·6H_2_O (238 mg, 1 mmol) in ethanol (4 ml) was added to a colourless solution containing *L* (250 mg, 1 mmol) in ethanol (20 ml) and stirred for a few minutes. A fraction of the total volume of the resulting blue solution (about 3 ml) was transferred into a test tube and left to evaporate slowly under ambient conditions. Within a few days, violet single crystals were harvested.

## 6. Refinement

Crystal data, data collection and structure refinement details are summarized in Table 2 ▸. The C-bound H atoms were placed in geometrically idealized positions (C—H = 0.95–1.00 Å) while those attached to O were positioned from a difference-Fourier map, then refined for a few cycles to ensure that reasonable displacement parameters could be achieved. Their coordinates were adjusted to give O—H = 0.87 Å. All hydrogen atoms were refined using a riding model with isotropic displacement parameters 1.2–1.5 times those of the parent atoms.

## Supplementary Material

Crystal structure: contains datablock(s) I. DOI: 10.1107/S2056989022003589/wm5636sup1.cif

Structure factors: contains datablock(s) I. DOI: 10.1107/S2056989022003589/wm5636Isup2.hkl

CCDC reference: 2163153

Additional supporting information:  crystallographic information; 3D view; checkCIF report

## Figures and Tables

**Table 1:** Hydrogen-bond geometry (Å, °)

*D*—H⋯*A*	*D*—H	H⋯*A*	*D*⋯*A*	*D*—H⋯*A*
O1—H1*A*⋯Cl1	0.84 (10)	2.37 (10)	3.194 (7)	166 (9)
O1—H1*B*⋯Cl4^i^	0.87 (10)	2.43 (10)	3.260 (7)	161 (9)

Symmetry code: (i) 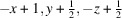 .

**Table 2:** Experimental details

Crystal data	
Chemical formula	[Co_2_Cl_4_(C_17_H_18_N_2_)_2_][CoCl_2_(C_17_H_18_N_2_)(H_2_O)]
*M*_r_	1158.50
Crystal system, space group	Orthorhombic, *P*2_1_2_1_2_1_
Temperature (K)	200
*a*, *b*, *c* (Å)	8.5470 (4), 22.0971 (9), 26.9407 (12)
*V* (Å^3^)	5088.1 (4)
*Z*	4
Radiation type	Cu *K*α
μ (mm^−1^)	10.82
Crystal size (mm)	0.21 × 0.11 × 0.05

Data collection	
Diffractometer	Stoe IPDS 2T
Absorption correction	Integration (*X-RED32*; Stoe, 2016 ▸)
*T*_min_, *T*_max_	0.176, 0.523
No. of measured, independent and observed [*I* > 2σ(*I*)] reflections	40552, 8979, 7084
*R*_int_	0.129
(sin θ/λ)_max_ (Å^−1^)	0.602

Refinement	
*R*[*F* ^2^ > 2σ(*F* ^2^)], *wR*(*F* ^2^), *S*	0.053, 0.138, 1.07
No. of reflections	8979
No. of parameters	617
H-atom treatment	H atoms treated by a mixture of independent and constrained refinement
Δρ_max_, Δρ_min_ (e Å^−3^)	0.63, −0.51
Absolute structure	Flack *x* determined using 2418 quotients [(*I* ^+^)−(*I* ^−^)]/[(*I* ^+^)+(*I* ^−^)] (Parsons *et al.*, 2013 ▸).
Absolute structure parameter	−0.042 (4)

Computer programs: *X-AREA* and *X-RED32* (Stoe, 2016 ▸), *SHELXT* (Sheldrick, 2015*a* ▸), *SHELXL* (Sheldrick, 2015*b* ▸), *OLEX2* (Dolomanov *et al.*, 2009 ▸), *PLATON* (Spek, 2020 ▸) and *publCIF* (Westrip, 2010 ▸).

## supplementary crystallographic information

### Crystal data

**Table:**

[Co_2_Cl_4_(C_17_H_18_N_2_)_2_][CoCl_2_(C_17_H_18_N_2_)(H_2_O)]	*D*_x_ = 1.512 Mg m^−^^3^
*M**_r_* = 1158.50	Cu *K*α radiation, λ = 1.54186 Å
Orthorhombic, *P*2_1_2_1_2_1_	Cell parameters from 32247 reflections
*a* = 8.5470 (4) Å	θ = 2.6–68.1°
*b* = 22.0971 (9) Å	µ = 10.82 mm^−^^1^
*c* = 26.9407 (12) Å	*T* = 200 K
*V* = 5088.1 (4) Å^3^	Prism, violet
*Z* = 4	0.21 × 0.11 × 0.05 mm
*F*(000) = 2380	

### Data collection

**Table:**

Stoe IPDS 2T diffractometer	8979 independent reflections
Radiation source: Genix-Cu, 3D, microfocus	7084 reflections with *I* > 2σ(*I*)
Multilayer optic monochromator	*R*_int_ = 0.129
Detector resolution: 6.67 pixels mm^-1^	θ_max_ = 68.1°, θ_min_ = 2.6°
rotation method, ω scans	*h* = −10→9
Absorption correction: integration (*X-Red32*; Stoe, 2016)	*k* = −25→26
*T*_min_ = 0.176, *T*_max_ = 0.523	*l* = −31→31
40552 measured reflections	

### Refinement

**Table:**

Refinement on *F*^2^	H atoms treated by a mixture of independent and constrained refinement
Least-squares matrix: full	*w* = 1/[σ^2^(*F*_o_^2^) + (0.0437*P*)^2^ + 12.2194*P*] where *P* = (*F*_o_^2^ + 2*F*_c_^2^)/3
*R*[*F*^2^ > 2σ(*F*^2^)] = 0.053	(Δ/σ)_max_ < 0.001
*wR*(*F*^2^) = 0.138	Δρ_max_ = 0.63 e Å^−^^3^
*S* = 1.07	Δρ_min_ = −0.51 e Å^−^^3^
8979 reflections	Extinction correction: SHELXL2017/1 (Sheldrick 2015b), Fc^*^=kFc[1+0.001xFc^2^λ^3^/sin(2θ)]^-1/4^
617 parameters	Extinction coefficient: 0.00083 (12)
0 restraints	Absolute structure: Flack *x* determined using 2418 quotients [(*I*^+^)-(*I*^-^)]/[(*I*^+^)+(*I*^-^)] (Parsons *et al.*, 2013).
Primary atom site location: dual	Absolute structure parameter: −0.042 (4)
Hydrogen site location: mixed	

### Special details

**Table:**

Geometry. All esds (except the esd in the dihedral angle between two l.s. planes) are estimated using the full covariance matrix. The cell esds are taken into account individually in the estimation of esds in distances, angles and torsion angles; correlations between esds in cell parameters are only used when they are defined by crystal symmetry. An approximate (isotropic) treatment of cell esds is used for estimating esds involving l.s. planes.

### Fractional atomic coordinates and isotropic or equivalent isotropic displacement parameters (Å^2^)

**Table:**

	*x*	*y*	*z*	*U*_iso_*/*U*_eq_	
Co1	0.59450 (15)	0.33142 (6)	0.21719 (5)	0.0261 (3)	
Co2	0.36326 (15)	0.20357 (6)	0.25146 (5)	0.0272 (3)	
Co3	0.34642 (15)	0.50356 (6)	0.35583 (5)	0.0276 (3)	
H1A	0.494 (12)	0.473 (4)	0.271 (4)	0.041*	
H1B	0.518 (12)	0.535 (5)	0.281 (4)	0.041*	
Cl1	0.4747 (3)	0.41998 (10)	0.19496 (8)	0.0416 (6)	
Cl2	0.5387 (2)	0.23709 (9)	0.18204 (8)	0.0335 (5)	
Cl3	0.4013 (3)	0.30157 (10)	0.27947 (8)	0.0361 (5)	
Cl4	0.4998 (3)	0.11977 (10)	0.27565 (8)	0.0373 (5)	
Cl5	0.4083 (3)	0.40985 (10)	0.38590 (9)	0.0416 (5)	
Cl6	0.4811 (3)	0.58759 (10)	0.38148 (8)	0.0398 (5)	
O1	0.5059 (7)	0.4990 (3)	0.2938 (2)	0.0365 (14)	
N1	0.7658 (8)	0.3446 (3)	0.2702 (3)	0.0269 (15)	
N2	0.7995 (8)	0.3417 (3)	0.1717 (3)	0.0263 (15)	
N3	0.1742 (8)	0.1839 (3)	0.2083 (2)	0.0279 (16)	
N4	0.1745 (8)	0.1953 (3)	0.3063 (2)	0.0272 (15)	
N5	0.1534 (8)	0.5088 (3)	0.3085 (3)	0.0286 (15)	
N6	0.1529 (7)	0.5121 (3)	0.4079 (2)	0.0247 (15)	
C1	0.7377 (10)	0.3510 (4)	0.3188 (3)	0.032 (2)	
H1	0.633294	0.348141	0.330610	0.038*	
C2	0.8577 (12)	0.3618 (4)	0.3522 (3)	0.041 (2)	
H2	0.835136	0.366735	0.386451	0.049*	
C3	1.0110 (11)	0.3653 (4)	0.3355 (3)	0.034 (2)	
H3	1.094813	0.371669	0.358029	0.041*	
C4	1.0386 (10)	0.3593 (4)	0.2854 (3)	0.032 (2)	
H4	1.142196	0.362691	0.272955	0.039*	
C5	0.9144 (10)	0.3483 (3)	0.2528 (3)	0.0258 (17)	
C6	0.9352 (8)	0.3422 (3)	0.1989 (3)	0.0223 (17)	
C7	1.0783 (10)	0.3370 (4)	0.1769 (3)	0.0303 (19)	
H7	1.170105	0.335479	0.196705	0.036*	
C8	1.0903 (10)	0.3339 (4)	0.1254 (3)	0.0313 (19)	
H8	1.189342	0.329634	0.109810	0.038*	
C9	0.9546 (10)	0.3371 (4)	0.0976 (3)	0.0292 (18)	
C10	0.8095 (9)	0.3408 (4)	0.1224 (3)	0.0251 (18)	
C11	0.6631 (10)	0.3451 (4)	0.0912 (3)	0.032 (2)	
H11A	0.605315	0.382354	0.100103	0.038*	
H11B	0.594716	0.310019	0.098448	0.038*	
C12	0.7014 (10)	0.3461 (4)	0.0360 (3)	0.033 (2)	
H12	0.609579	0.350497	0.013238	0.040*	
C13	0.8383 (11)	0.3905 (4)	0.0265 (3)	0.037 (2)	
H13A	0.840412	0.425698	0.049239	0.044*	
H13B	0.848812	0.403466	−0.008566	0.044*	
C14	0.9509 (10)	0.3382 (4)	0.0417 (3)	0.033 (2)	
H14	1.054115	0.336437	0.024254	0.040*	
C15	0.8196 (11)	0.2943 (4)	0.0222 (3)	0.034 (2)	
C16	0.8006 (11)	0.2322 (4)	0.0473 (3)	0.036 (2)	
H16A	0.708187	0.211769	0.033864	0.054*	
H16B	0.787762	0.237847	0.083197	0.054*	
H16C	0.893754	0.207563	0.040997	0.054*	
C17	0.8308 (12)	0.2847 (5)	−0.0338 (3)	0.044 (2)	
H17A	0.918498	0.257568	−0.041212	0.066*	
H17B	0.847684	0.323695	−0.050272	0.066*	
H17C	0.733323	0.266590	−0.045969	0.066*	
C18	0.1847 (10)	0.1732 (4)	0.1592 (3)	0.0292 (19)	
H18	0.283740	0.176303	0.143423	0.035*	
C19	0.0552 (10)	0.1578 (4)	0.1312 (3)	0.034 (2)	
H19	0.066389	0.149282	0.096842	0.041*	
C20	−0.0916 (10)	0.1547 (4)	0.1534 (3)	0.033 (2)	
H20	−0.182621	0.145898	0.134516	0.040*	
C21	−0.1003 (10)	0.1649 (4)	0.2038 (3)	0.0318 (19)	
H21	−0.197951	0.161697	0.220489	0.038*	
C22	0.0331 (9)	0.1797 (4)	0.2302 (3)	0.0264 (18)	
C23	0.0307 (9)	0.1907 (4)	0.2846 (3)	0.0274 (18)	
C24	−0.1050 (10)	0.1930 (4)	0.3122 (3)	0.034 (2)	
H24	−0.203972	0.188426	0.296598	0.041*	
C25	−0.0950 (11)	0.2022 (4)	0.3632 (3)	0.038 (2)	
H25	−0.187522	0.205081	0.382585	0.045*	
C26	0.0491 (10)	0.2071 (4)	0.3854 (3)	0.0314 (19)	
C27	0.1835 (9)	0.2034 (4)	0.3552 (3)	0.0273 (18)	
C28	0.3411 (11)	0.2064 (5)	0.3802 (3)	0.040 (2)	
H28A	0.401156	0.240988	0.366747	0.048*	
H28B	0.400354	0.168884	0.373056	0.048*	
C29	0.3229 (12)	0.2137 (4)	0.4364 (3)	0.040 (2)	
H29	0.423458	0.217251	0.455186	0.048*	
C30	0.2034 (12)	0.2649 (5)	0.4465 (4)	0.045 (2)	
H30A	0.201003	0.296958	0.420834	0.054*	
H30B	0.209118	0.282349	0.480339	0.054*	
C31	0.0764 (12)	0.2152 (4)	0.4400 (3)	0.040 (2)	
H31	−0.019252	0.219355	0.461137	0.047*	
C32	0.2033 (12)	0.1683 (5)	0.4587 (4)	0.042 (2)	
C33	0.1953 (14)	0.1045 (5)	0.4379 (4)	0.053 (3)	
H33A	0.291683	0.082708	0.446264	0.079*	
H33B	0.183686	0.106261	0.401750	0.079*	
H33C	0.105422	0.083267	0.452311	0.079*	
C34	0.2119 (15)	0.1650 (6)	0.5151 (4)	0.060 (3)	
H34A	0.122910	0.141598	0.527733	0.091*	
H34B	0.208541	0.205985	0.528978	0.091*	
H34C	0.309717	0.145238	0.525017	0.091*	
C35	0.1575 (11)	0.5104 (4)	0.2590 (3)	0.035 (2)	
H35	0.256420	0.508368	0.243038	0.043*	
C36	0.0247 (11)	0.5150 (4)	0.2297 (3)	0.040 (2)	
H36	0.032768	0.515822	0.194525	0.048*	
C37	−0.1175 (11)	0.5183 (5)	0.2523 (4)	0.041 (2)	
H37	−0.210136	0.521652	0.233001	0.049*	
C38	−0.1261 (11)	0.5167 (4)	0.3032 (4)	0.039 (2)	
H38	−0.224616	0.519085	0.319403	0.047*	
C39	0.0098 (9)	0.5117 (4)	0.3307 (3)	0.0284 (18)	
C40	0.0108 (9)	0.5097 (4)	0.3862 (3)	0.0278 (18)	
C41	−0.1264 (10)	0.5042 (4)	0.4130 (3)	0.0321 (19)	
H41	−0.224508	0.501035	0.396664	0.039*	
C42	−0.1172 (10)	0.5036 (4)	0.4646 (3)	0.0318 (19)	
H42	−0.209570	0.499709	0.483986	0.038*	
C43	0.0268 (10)	0.5086 (4)	0.4875 (3)	0.0291 (18)	
C44	0.1613 (10)	0.5124 (4)	0.4574 (3)	0.0291 (19)	
C45	0.3188 (10)	0.5155 (4)	0.4826 (3)	0.036 (2)	
H45A	0.373290	0.553019	0.472336	0.043*	
H45B	0.383375	0.480620	0.472063	0.043*	
C46	0.3010 (11)	0.5148 (4)	0.5391 (3)	0.036 (2)	
H46	0.400691	0.518064	0.558356	0.043*	
C47	0.1712 (11)	0.5602 (4)	0.5540 (3)	0.035 (2)	
H47A	0.162442	0.595626	0.531732	0.042*	
H47B	0.174588	0.572696	0.589298	0.042*	
C48	0.0517 (10)	0.5089 (4)	0.5428 (3)	0.033 (2)	
H48	−0.044342	0.507588	0.564008	0.040*	
C49	0.1861 (12)	0.4632 (4)	0.5563 (3)	0.034 (2)	
C50	0.1942 (13)	0.4021 (4)	0.5297 (4)	0.045 (2)	
H50A	0.292556	0.381758	0.538188	0.067*	
H50B	0.189308	0.408512	0.493756	0.067*	
H50C	0.105855	0.376814	0.540143	0.067*	
C51	0.1961 (13)	0.4523 (5)	0.6124 (4)	0.048 (3)	
H51A	0.107222	0.427388	0.622993	0.072*	
H51B	0.193296	0.491166	0.629808	0.072*	
H51C	0.293984	0.431247	0.620174	0.072*	

### Atomic displacement parameters (Å^2^)

**Table:**

	*U*^11^	*U*^22^	*U*^33^	*U*^12^	*U*^13^	*U*^23^
Co1	0.0219 (7)	0.0243 (7)	0.0321 (7)	0.0015 (5)	−0.0018 (6)	−0.0016 (6)
Co2	0.0223 (7)	0.0276 (7)	0.0317 (7)	0.0009 (6)	−0.0020 (6)	0.0015 (6)
Co3	0.0202 (7)	0.0290 (7)	0.0335 (7)	0.0008 (6)	−0.0011 (6)	−0.0008 (6)
Cl1	0.0492 (14)	0.0341 (11)	0.0416 (12)	0.0167 (10)	−0.0021 (11)	0.0008 (9)
Cl2	0.0325 (11)	0.0299 (10)	0.0382 (11)	−0.0058 (9)	0.0089 (9)	−0.0055 (9)
Cl3	0.0336 (11)	0.0342 (11)	0.0404 (11)	−0.0040 (9)	0.0092 (10)	−0.0076 (9)
Cl4	0.0370 (12)	0.0346 (11)	0.0402 (12)	0.0098 (9)	−0.0051 (10)	0.0038 (9)
Cl5	0.0469 (13)	0.0316 (11)	0.0463 (13)	0.0114 (10)	0.0015 (11)	0.0020 (10)
Cl6	0.0375 (12)	0.0360 (11)	0.0460 (13)	−0.0144 (10)	−0.0018 (10)	−0.0004 (10)
O1	0.027 (3)	0.040 (4)	0.042 (4)	0.001 (3)	0.004 (3)	−0.006 (3)
N1	0.022 (4)	0.029 (4)	0.029 (4)	−0.004 (3)	−0.001 (3)	0.002 (3)
N2	0.024 (4)	0.023 (4)	0.032 (4)	−0.002 (3)	0.000 (3)	−0.001 (3)
N3	0.031 (4)	0.023 (3)	0.030 (4)	0.002 (3)	0.001 (3)	0.001 (3)
N4	0.024 (4)	0.027 (4)	0.031 (4)	0.002 (3)	0.001 (3)	0.004 (3)
N5	0.027 (4)	0.026 (4)	0.033 (4)	0.000 (3)	0.003 (3)	0.001 (3)
N6	0.012 (3)	0.029 (4)	0.033 (4)	0.004 (3)	0.003 (3)	0.000 (3)
C1	0.031 (5)	0.037 (5)	0.028 (5)	−0.005 (4)	−0.003 (4)	−0.002 (4)
C2	0.052 (6)	0.044 (5)	0.027 (5)	−0.008 (5)	−0.004 (5)	0.001 (4)
C3	0.037 (5)	0.027 (4)	0.037 (5)	−0.005 (4)	−0.017 (4)	−0.005 (4)
C4	0.023 (4)	0.034 (5)	0.039 (5)	−0.003 (4)	−0.010 (4)	−0.005 (4)
C5	0.027 (4)	0.017 (4)	0.033 (4)	−0.001 (3)	−0.003 (4)	−0.002 (3)
C6	0.010 (4)	0.022 (4)	0.035 (4)	−0.001 (3)	−0.002 (3)	0.000 (3)
C7	0.020 (4)	0.031 (4)	0.039 (5)	−0.001 (4)	−0.006 (4)	−0.003 (4)
C8	0.016 (4)	0.035 (5)	0.043 (5)	−0.006 (4)	0.001 (4)	−0.003 (4)
C9	0.025 (4)	0.029 (4)	0.034 (5)	−0.002 (4)	−0.001 (4)	−0.003 (4)
C10	0.017 (4)	0.027 (4)	0.031 (4)	0.009 (3)	0.000 (3)	−0.002 (4)
C11	0.024 (4)	0.042 (5)	0.029 (4)	0.002 (4)	−0.001 (4)	0.003 (4)
C12	0.030 (5)	0.034 (5)	0.034 (5)	0.011 (4)	−0.009 (4)	0.005 (4)
C13	0.034 (5)	0.040 (5)	0.036 (5)	0.002 (4)	0.005 (4)	0.013 (4)
C14	0.025 (5)	0.039 (5)	0.035 (5)	0.005 (4)	0.006 (4)	0.003 (4)
C15	0.034 (5)	0.037 (5)	0.030 (4)	0.008 (4)	−0.007 (4)	0.003 (4)
C16	0.038 (5)	0.030 (5)	0.039 (5)	−0.002 (4)	−0.004 (4)	−0.001 (4)
C17	0.039 (6)	0.057 (7)	0.036 (5)	0.008 (5)	−0.003 (4)	0.004 (5)
C18	0.025 (4)	0.028 (4)	0.034 (5)	−0.005 (4)	−0.001 (4)	0.001 (4)
C19	0.030 (5)	0.035 (5)	0.037 (5)	−0.006 (4)	−0.002 (4)	−0.001 (4)
C20	0.026 (4)	0.029 (5)	0.045 (5)	−0.005 (4)	−0.011 (4)	0.003 (4)
C21	0.017 (4)	0.043 (5)	0.036 (5)	−0.004 (4)	0.000 (4)	0.005 (4)
C22	0.018 (4)	0.029 (4)	0.032 (4)	−0.001 (3)	0.003 (4)	0.007 (3)
C23	0.017 (4)	0.031 (4)	0.035 (5)	−0.001 (3)	−0.005 (4)	0.004 (4)
C24	0.021 (4)	0.037 (5)	0.044 (5)	0.004 (4)	0.005 (4)	0.000 (4)
C25	0.029 (5)	0.040 (5)	0.044 (5)	0.003 (4)	0.009 (4)	0.004 (4)
C26	0.031 (5)	0.025 (4)	0.039 (5)	0.004 (4)	0.000 (4)	0.008 (4)
C27	0.022 (4)	0.032 (4)	0.028 (4)	0.003 (4)	0.002 (4)	0.001 (4)
C28	0.026 (5)	0.057 (6)	0.035 (5)	0.000 (4)	−0.006 (4)	0.002 (5)
C29	0.041 (6)	0.047 (6)	0.031 (5)	0.004 (5)	−0.002 (4)	0.000 (4)
C30	0.055 (7)	0.042 (6)	0.040 (6)	0.000 (5)	−0.001 (5)	0.000 (5)
C31	0.045 (6)	0.038 (5)	0.036 (5)	0.012 (4)	0.005 (4)	−0.002 (4)
C32	0.045 (6)	0.044 (6)	0.037 (5)	0.005 (5)	0.004 (5)	0.007 (5)
C33	0.067 (8)	0.038 (6)	0.052 (6)	0.013 (5)	−0.010 (6)	0.011 (5)
C34	0.063 (8)	0.078 (8)	0.041 (6)	0.004 (7)	0.000 (6)	0.015 (6)
C35	0.027 (5)	0.046 (6)	0.033 (5)	0.004 (4)	0.004 (4)	0.003 (4)
C36	0.037 (5)	0.053 (6)	0.029 (5)	0.003 (5)	−0.005 (4)	0.001 (4)
C37	0.026 (5)	0.060 (6)	0.037 (5)	−0.003 (4)	−0.004 (4)	0.000 (5)
C38	0.021 (5)	0.053 (6)	0.044 (5)	0.000 (4)	−0.002 (4)	0.005 (4)
C39	0.018 (4)	0.029 (4)	0.038 (5)	0.005 (3)	−0.001 (4)	0.003 (4)
C40	0.020 (4)	0.025 (4)	0.038 (5)	0.004 (3)	0.002 (4)	0.000 (4)
C41	0.023 (4)	0.032 (5)	0.042 (5)	0.002 (4)	0.002 (4)	0.000 (4)
C42	0.024 (4)	0.028 (4)	0.043 (5)	−0.004 (4)	0.014 (4)	−0.004 (4)
C43	0.028 (4)	0.024 (4)	0.036 (5)	−0.003 (4)	0.009 (4)	−0.002 (4)
C44	0.027 (4)	0.027 (4)	0.033 (5)	0.003 (4)	0.002 (4)	0.001 (4)
C45	0.024 (5)	0.045 (5)	0.038 (5)	−0.003 (4)	−0.001 (4)	−0.001 (4)
C46	0.040 (5)	0.035 (5)	0.033 (5)	−0.003 (4)	0.001 (4)	0.000 (4)
C47	0.035 (5)	0.029 (4)	0.040 (5)	−0.006 (4)	0.003 (4)	−0.010 (4)
C48	0.027 (5)	0.035 (5)	0.037 (5)	−0.007 (4)	0.005 (4)	−0.007 (4)
C49	0.044 (6)	0.024 (4)	0.033 (5)	−0.001 (4)	0.002 (4)	−0.002 (4)
C50	0.055 (7)	0.027 (5)	0.052 (6)	0.004 (4)	−0.002 (5)	−0.008 (4)
C51	0.060 (7)	0.047 (6)	0.037 (5)	−0.005 (5)	0.000 (5)	−0.001 (5)

### Geometric parameters (Å, º)

**Table:**

Co1—Cl1	2.288 (2)	C20—H20	0.9500
Co1—Cl2	2.339 (2)	C20—C21	1.379 (12)
Co1—Cl3	2.445 (2)	C21—H21	0.9500
Co1—N1	2.066 (7)	C21—C22	1.382 (11)
Co1—N2	2.151 (7)	C22—C23	1.486 (11)
Co2—Cl2	2.509 (2)	C23—C24	1.379 (11)
Co2—Cl3	2.316 (2)	C24—H24	0.9500
Co2—Cl4	2.284 (2)	C24—C25	1.392 (12)
Co2—N3	2.037 (7)	C25—H25	0.9500
Co2—N4	2.195 (7)	C25—C26	1.374 (12)
Co3—Cl5	2.286 (3)	C26—C27	1.411 (11)
Co3—Cl6	2.291 (2)	C26—C31	1.501 (12)
Co3—O1	2.160 (6)	C27—C28	1.508 (12)
Co3—N5	2.089 (7)	C28—H28A	0.9900
Co3—N6	2.178 (6)	C28—H28B	0.9900
O1—H1A	0.84 (10)	C28—C29	1.531 (12)
O1—H1B	0.87 (10)	C29—H29	1.0000
N1—C1	1.338 (11)	C29—C30	1.549 (14)
N1—C5	1.356 (10)	C29—C32	1.553 (14)
N2—C6	1.373 (10)	C30—H30A	0.9900
N2—C10	1.332 (10)	C30—H30B	0.9900
N3—C18	1.347 (10)	C30—C31	1.555 (14)
N3—C22	1.345 (10)	C31—H31	1.0000
N4—C23	1.365 (10)	C31—C32	1.581 (13)
N4—C27	1.330 (10)	C32—C33	1.519 (14)
N5—C35	1.334 (10)	C32—C34	1.525 (13)
N5—C39	1.366 (10)	C33—H33A	0.9800
N6—C40	1.350 (10)	C33—H33B	0.9800
N6—C44	1.333 (10)	C33—H33C	0.9800
C1—H1	0.9500	C34—H34A	0.9800
C1—C2	1.385 (13)	C34—H34B	0.9800
C2—H2	0.9500	C34—H34C	0.9800
C2—C3	1.387 (14)	C35—H35	0.9500
C3—H3	0.9500	C35—C36	1.386 (13)
C3—C4	1.375 (12)	C36—H36	0.9500
C4—H4	0.9500	C36—C37	1.361 (13)
C4—C5	1.399 (12)	C37—H37	0.9500
C5—C6	1.469 (11)	C37—C38	1.374 (13)
C6—C7	1.364 (11)	C38—H38	0.9500
C7—H7	0.9500	C38—C39	1.381 (12)
C7—C8	1.391 (12)	C39—C40	1.496 (11)
C8—H8	0.9500	C40—C41	1.383 (11)
C8—C9	1.383 (12)	C41—H41	0.9500
C9—C10	1.411 (11)	C41—C42	1.392 (12)
C9—C14	1.506 (12)	C42—H42	0.9500
C10—C11	1.509 (11)	C42—C43	1.381 (12)
C11—H11A	0.9900	C43—C44	1.410 (11)
C11—H11B	0.9900	C43—C48	1.507 (12)
C11—C12	1.524 (12)	C44—C45	1.509 (12)
C12—H12	1.0000	C45—H45A	0.9900
C12—C13	1.549 (13)	C45—H45B	0.9900
C12—C15	1.571 (12)	C45—C46	1.531 (12)
C13—H13A	0.9900	C46—H46	1.0000
C13—H13B	0.9900	C46—C47	1.548 (13)
C13—C14	1.560 (12)	C46—C49	1.575 (13)
C14—H14	1.0000	C47—H47A	0.9900
C14—C15	1.573 (13)	C47—H47B	0.9900
C15—C16	1.538 (12)	C47—C48	1.556 (11)
C15—C17	1.527 (12)	C48—H48	1.0000
C16—H16A	0.9800	C48—C49	1.573 (13)
C16—H16B	0.9800	C49—C50	1.530 (12)
C16—H16C	0.9800	C49—C51	1.531 (13)
C17—H17A	0.9800	C50—H50A	0.9800
C17—H17B	0.9800	C50—H50B	0.9800
C17—H17C	0.9800	C50—H50C	0.9800
C18—H18	0.9500	C51—H51A	0.9800
C18—C19	1.382 (12)	C51—H51B	0.9800
C19—H19	0.9500	C51—H51C	0.9800
C19—C20	1.392 (12)		
			
Cl1—Co1—Cl2	124.40 (10)	C20—C21—C22	120.0 (8)
Cl1—Co1—Cl3	96.21 (9)	C22—C21—H21	120.0
Cl2—Co1—Cl3	84.24 (8)	N3—C22—C21	122.1 (8)
N1—Co1—Cl1	112.2 (2)	N3—C22—C23	115.6 (7)
N1—Co1—Cl2	123.4 (2)	C21—C22—C23	122.3 (7)
N1—Co1—Cl3	92.4 (2)	N4—C23—C22	115.0 (7)
N1—Co1—N2	78.5 (3)	N4—C23—C24	121.6 (7)
N2—Co1—Cl1	97.2 (2)	C24—C23—C22	123.3 (7)
N2—Co1—Cl2	91.72 (19)	C23—C24—H24	120.4
N2—Co1—Cl3	166.0 (2)	C23—C24—C25	119.1 (8)
Cl3—Co2—Cl2	83.28 (8)	C25—C24—H24	120.4
Cl4—Co2—Cl2	98.44 (9)	C24—C25—H25	120.1
Cl4—Co2—Cl3	126.39 (10)	C26—C25—C24	119.8 (8)
N3—Co2—Cl2	96.4 (2)	C26—C25—H25	120.1
N3—Co2—Cl3	119.8 (2)	C25—C26—C27	118.3 (8)
N3—Co2—Cl4	113.3 (2)	C25—C26—C31	125.2 (8)
N3—Co2—N4	77.5 (3)	C27—C26—C31	116.5 (8)
N4—Co2—Cl2	164.91 (19)	N4—C27—C26	122.1 (7)
N4—Co2—Cl3	87.81 (19)	N4—C27—C28	120.0 (7)
N4—Co2—Cl4	96.65 (19)	C26—C27—C28	117.8 (7)
Cl5—Co3—Cl6	120.73 (10)	C27—C28—H28A	109.5
O1—Co3—Cl5	94.9 (2)	C27—C28—H28B	109.5
O1—Co3—Cl6	87.4 (2)	C27—C28—C29	110.9 (8)
O1—Co3—N6	169.3 (3)	H28A—C28—H28B	108.0
N5—Co3—Cl5	116.7 (2)	C29—C28—H28A	109.5
N5—Co3—Cl6	122.4 (2)	C29—C28—H28B	109.5
N5—Co3—O1	91.6 (3)	C28—C29—H29	114.9
N5—Co3—N6	77.8 (3)	C28—C29—C30	108.6 (8)
N6—Co3—Cl5	91.49 (19)	C28—C29—C32	112.4 (8)
N6—Co3—Cl6	96.71 (19)	C30—C29—H29	114.9
Co1—Cl2—Co2	94.77 (8)	C30—C29—C32	88.3 (8)
Co2—Cl3—Co1	97.09 (9)	C32—C29—H29	114.9
Co3—O1—H1A	121 (7)	C29—C30—H30A	114.4
Co3—O1—H1B	109 (7)	C29—C30—H30B	114.4
H1A—O1—H1B	111 (9)	C29—C30—C31	85.6 (7)
C1—N1—Co1	124.3 (6)	H30A—C30—H30B	111.5
C1—N1—C5	119.9 (7)	C31—C30—H30A	114.4
C5—N1—Co1	115.7 (5)	C31—C30—H30B	114.4
C6—N2—Co1	112.6 (5)	C26—C31—C30	107.6 (8)
C10—N2—Co1	128.3 (5)	C26—C31—H31	116.2
C10—N2—C6	118.7 (7)	C26—C31—C32	109.9 (7)
C18—N3—Co2	123.0 (6)	C30—C31—H31	116.2
C22—N3—Co2	118.5 (5)	C30—C31—C32	87.1 (7)
C22—N3—C18	118.5 (7)	C32—C31—H31	116.2
C23—N4—Co2	112.3 (5)	C29—C32—C31	84.6 (7)
C27—N4—Co2	127.7 (5)	C33—C32—C29	119.2 (9)
C27—N4—C23	119.1 (7)	C33—C32—C31	117.4 (9)
C35—N5—Co3	126.2 (6)	C33—C32—C34	109.0 (9)
C35—N5—C39	117.4 (7)	C34—C32—C29	112.6 (9)
C39—N5—Co3	116.4 (5)	C34—C32—C31	112.4 (8)
C40—N6—Co3	113.6 (5)	C32—C33—H33A	109.5
C44—N6—Co3	127.1 (6)	C32—C33—H33B	109.5
C44—N6—C40	118.9 (7)	C32—C33—H33C	109.5
N1—C1—H1	119.3	H33A—C33—H33B	109.5
N1—C1—C2	121.4 (8)	H33A—C33—H33C	109.5
C2—C1—H1	119.3	H33B—C33—H33C	109.5
C1—C2—H2	120.1	C32—C34—H34A	109.5
C1—C2—C3	119.9 (9)	C32—C34—H34B	109.5
C3—C2—H2	120.1	C32—C34—H34C	109.5
C2—C3—H3	120.8	H34A—C34—H34B	109.5
C4—C3—C2	118.3 (8)	H34A—C34—H34C	109.5
C4—C3—H3	120.8	H34B—C34—H34C	109.5
C3—C4—H4	119.9	N5—C35—H35	118.3
C3—C4—C5	120.1 (8)	N5—C35—C36	123.4 (8)
C5—C4—H4	119.9	C36—C35—H35	118.3
N1—C5—C4	120.3 (8)	C35—C36—H36	120.7
N1—C5—C6	116.7 (7)	C37—C36—C35	118.7 (8)
C4—C5—C6	123.0 (8)	C37—C36—H36	120.7
N2—C6—C5	115.2 (7)	C36—C37—H37	120.2
C7—C6—N2	121.6 (7)	C36—C37—C38	119.5 (9)
C7—C6—C5	123.2 (7)	C38—C37—H37	120.2
C6—C7—H7	119.9	C37—C38—H38	120.3
C6—C7—C8	120.3 (8)	C37—C38—C39	119.5 (8)
C8—C7—H7	119.9	C39—C38—H38	120.3
C7—C8—H8	120.8	N5—C39—C38	121.6 (8)
C9—C8—C7	118.5 (8)	N5—C39—C40	115.5 (7)
C9—C8—H8	120.8	C38—C39—C40	122.8 (8)
C8—C9—C10	118.9 (8)	N6—C40—C39	116.0 (7)
C8—C9—C14	124.1 (8)	N6—C40—C41	122.6 (8)
C10—C9—C14	117.0 (7)	C41—C40—C39	121.4 (8)
N2—C10—C9	122.0 (7)	C40—C41—H41	120.8
N2—C10—C11	120.1 (7)	C40—C41—C42	118.4 (8)
C9—C10—C11	118.0 (7)	C42—C41—H41	120.8
C10—C11—H11A	109.3	C41—C42—H42	120.2
C10—C11—H11B	109.3	C43—C42—C41	119.6 (8)
C10—C11—C12	111.5 (7)	C43—C42—H42	120.2
H11A—C11—H11B	108.0	C42—C43—C44	118.4 (8)
C12—C11—H11A	109.3	C42—C43—C48	124.6 (7)
C12—C11—H11B	109.3	C44—C43—C48	117.0 (8)
C11—C12—H12	115.5	N6—C44—C43	122.0 (8)
C11—C12—C13	109.5 (7)	N6—C44—C45	119.9 (7)
C11—C12—C15	110.9 (7)	C43—C44—C45	118.1 (7)
C13—C12—H12	115.5	C44—C45—H45A	109.4
C13—C12—C15	86.4 (7)	C44—C45—H45B	109.4
C15—C12—H12	115.5	C44—C45—C46	111.0 (7)
C12—C13—H13A	114.1	H45A—C45—H45B	108.0
C12—C13—H13B	114.1	C46—C45—H45A	109.4
C12—C13—C14	87.3 (6)	C46—C45—H45B	109.4
H13A—C13—H13B	111.3	C45—C46—H46	115.5
C14—C13—H13A	114.1	C45—C46—C47	108.8 (8)
C14—C13—H13B	114.1	C45—C46—C49	111.2 (7)
C9—C14—C13	106.7 (7)	C47—C46—H46	115.5
C9—C14—H14	116.7	C47—C46—C49	86.9 (7)
C9—C14—C15	109.8 (7)	C49—C46—H46	115.5
C13—C14—H14	116.7	C46—C47—H47A	114.1
C13—C14—C15	86.0 (7)	C46—C47—H47B	114.1
C15—C14—H14	116.7	C46—C47—C48	87.1 (7)
C12—C15—C14	86.0 (7)	H47A—C47—H47B	111.3
C16—C15—C12	118.5 (8)	C48—C47—H47A	114.1
C16—C15—C14	118.5 (7)	C48—C47—H47B	114.1
C17—C15—C12	112.0 (7)	C43—C48—C47	106.8 (7)
C17—C15—C14	111.8 (8)	C43—C48—H48	116.7
C17—C15—C16	108.5 (8)	C43—C48—C49	109.2 (7)
C15—C16—H16A	109.5	C47—C48—H48	116.7
C15—C16—H16B	109.5	C47—C48—C49	86.7 (7)
C15—C16—H16C	109.5	C49—C48—H48	116.7
H16A—C16—H16B	109.5	C48—C49—C46	85.5 (6)
H16A—C16—H16C	109.5	C50—C49—C46	118.3 (8)
H16B—C16—H16C	109.5	C50—C49—C48	119.4 (8)
C15—C17—H17A	109.5	C50—C49—C51	108.7 (8)
C15—C17—H17B	109.5	C51—C49—C46	111.7 (8)
C15—C17—H17C	109.5	C51—C49—C48	111.7 (8)
H17A—C17—H17B	109.5	C49—C50—H50A	109.5
H17A—C17—H17C	109.5	C49—C50—H50B	109.5
H17B—C17—H17C	109.5	C49—C50—H50C	109.5
N3—C18—H18	119.1	H50A—C50—H50B	109.5
N3—C18—C19	121.7 (8)	H50A—C50—H50C	109.5
C19—C18—H18	119.1	H50B—C50—H50C	109.5
C18—C19—H19	120.0	C49—C51—H51A	109.5
C18—C19—C20	120.0 (8)	C49—C51—H51B	109.5
C20—C19—H19	120.0	C49—C51—H51C	109.5
C19—C20—H20	121.2	H51A—C51—H51B	109.5
C21—C20—C19	117.6 (8)	H51A—C51—H51C	109.5
C21—C20—H20	121.2	H51B—C51—H51C	109.5
C20—C21—H21	120.0		
			
Co1—N1—C1—C2	−177.8 (7)	C20—C21—C22—N3	−0.9 (13)
Co1—N1—C5—C4	177.8 (6)	C20—C21—C22—C23	−179.6 (8)
Co1—N1—C5—C6	−0.4 (9)	C21—C22—C23—N4	170.0 (8)
Co1—N2—C6—C5	−12.1 (8)	C21—C22—C23—C24	−6.6 (13)
Co1—N2—C6—C7	167.6 (6)	C22—N3—C18—C19	−0.4 (12)
Co1—N2—C10—C9	−168.1 (6)	C22—C23—C24—C25	178.5 (8)
Co1—N2—C10—C11	13.1 (11)	C23—N4—C27—C26	0.4 (12)
Co2—N3—C18—C19	177.6 (6)	C23—N4—C27—C28	177.9 (8)
Co2—N3—C22—C21	−178.2 (6)	C23—C24—C25—C26	−1.8 (14)
Co2—N3—C22—C23	0.6 (9)	C24—C25—C26—C27	0.9 (13)
Co2—N4—C23—C22	11.9 (9)	C24—C25—C26—C31	−178.1 (8)
Co2—N4—C23—C24	−171.5 (7)	C25—C26—C27—N4	−0.1 (13)
Co2—N4—C27—C26	168.7 (6)	C25—C26—C27—C28	−177.7 (8)
Co2—N4—C27—C28	−13.8 (12)	C25—C26—C31—C30	−135.5 (9)
Co3—N5—C35—C36	−178.8 (7)	C25—C26—C31—C32	131.2 (9)
Co3—N5—C39—C38	178.5 (7)	C26—C27—C28—C29	−0.5 (12)
Co3—N5—C39—C40	−1.0 (9)	C26—C31—C32—C29	79.7 (8)
Co3—N6—C40—C39	−9.5 (9)	C26—C31—C32—C33	−40.5 (12)
Co3—N6—C40—C41	169.1 (7)	C26—C31—C32—C34	−168.0 (9)
Co3—N6—C44—C43	−169.7 (6)	C27—N4—C23—C22	−178.0 (7)
Co3—N6—C44—C45	9.0 (11)	C27—N4—C23—C24	−1.4 (12)
N1—C1—C2—C3	−0.8 (14)	C27—C26—C31—C30	45.5 (10)
N1—C5—C6—N2	8.6 (10)	C27—C26—C31—C32	−47.8 (11)
N1—C5—C6—C7	−171.1 (8)	C27—C28—C29—C30	−48.1 (11)
N2—C6—C7—C8	3.0 (13)	C27—C28—C29—C32	47.9 (11)
N2—C10—C11—C12	177.4 (7)	C28—C29—C30—C31	84.5 (8)
N3—C18—C19—C20	1.9 (13)	C28—C29—C32—C31	−81.3 (9)
N3—C22—C23—N4	−8.8 (10)	C28—C29—C32—C33	37.2 (12)
N3—C22—C23—C24	174.6 (8)	C28—C29—C32—C34	166.6 (9)
N4—C23—C24—C25	2.1 (13)	C29—C30—C31—C26	−81.9 (8)
N4—C27—C28—C29	−178.1 (8)	C29—C30—C31—C32	28.0 (7)
N5—C35—C36—C37	0.3 (15)	C30—C29—C32—C31	28.1 (7)
N5—C39—C40—N6	7.2 (11)	C30—C29—C32—C33	146.5 (9)
N5—C39—C40—C41	−171.4 (8)	C30—C29—C32—C34	−84.0 (9)
N6—C40—C41—C42	2.5 (12)	C30—C31—C32—C29	−28.0 (7)
N6—C44—C45—C46	−179.6 (7)	C30—C31—C32—C33	−148.1 (9)
C1—N1—C5—C4	−0.5 (12)	C30—C31—C32—C34	84.3 (10)
C1—N1—C5—C6	−178.6 (7)	C31—C26—C27—N4	178.9 (8)
C1—C2—C3—C4	1.5 (13)	C31—C26—C27—C28	1.4 (11)
C2—C3—C4—C5	−1.7 (12)	C32—C29—C30—C31	−28.6 (7)
C3—C4—C5—N1	1.3 (12)	C35—N5—C39—C38	−0.6 (12)
C3—C4—C5—C6	179.3 (8)	C35—N5—C39—C40	179.9 (7)
C4—C5—C6—N2	−169.4 (7)	C35—C36—C37—C38	−0.3 (15)
C4—C5—C6—C7	10.8 (12)	C36—C37—C38—C39	−0.1 (15)
C5—N1—C1—C2	0.3 (13)	C37—C38—C39—N5	0.6 (14)
C5—C6—C7—C8	−177.3 (7)	C37—C38—C39—C40	−180.0 (9)
C6—N2—C10—C9	3.4 (12)	C38—C39—C40—N6	−172.3 (8)
C6—N2—C10—C11	−175.4 (7)	C38—C39—C40—C41	9.2 (13)
C6—C7—C8—C9	0.9 (13)	C39—N5—C35—C36	0.1 (14)
C7—C8—C9—C10	−2.6 (12)	C39—C40—C41—C42	−179.0 (7)
C7—C8—C9—C14	176.0 (8)	C40—N6—C44—C43	1.8 (12)
C8—C9—C10—N2	0.4 (12)	C40—N6—C44—C45	−179.6 (8)
C8—C9—C10—C11	179.2 (8)	C40—C41—C42—C43	0.3 (12)
C8—C9—C14—C13	−132.5 (9)	C41—C42—C43—C44	−1.9 (12)
C8—C9—C14—C15	135.7 (9)	C41—C42—C43—C48	179.5 (8)
C9—C10—C11—C12	−1.4 (11)	C42—C43—C44—N6	0.9 (12)
C9—C14—C15—C12	78.5 (7)	C42—C43—C44—C45	−177.8 (8)
C9—C14—C15—C16	−42.0 (10)	C42—C43—C48—C47	−135.9 (9)
C9—C14—C15—C17	−169.3 (7)	C42—C43—C48—C49	131.6 (9)
C10—N2—C6—C5	175.1 (7)	C43—C44—C45—C46	−0.9 (11)
C10—N2—C6—C7	−5.1 (12)	C43—C48—C49—C46	79.3 (7)
C10—C9—C14—C13	46.1 (10)	C43—C48—C49—C50	−40.7 (11)
C10—C9—C14—C15	−45.6 (10)	C43—C48—C49—C51	−169.1 (7)
C10—C11—C12—C13	−45.3 (10)	C44—N6—C40—C39	177.9 (7)
C10—C11—C12—C15	48.3 (10)	C44—N6—C40—C41	−3.5 (12)
C11—C12—C13—C14	82.7 (8)	C44—C43—C48—C47	45.4 (10)
C11—C12—C15—C14	−81.4 (8)	C44—C43—C48—C49	−47.0 (10)
C11—C12—C15—C16	39.0 (11)	C44—C45—C46—C47	−46.5 (10)
C11—C12—C15—C17	166.7 (8)	C44—C45—C46—C49	47.6 (10)
C12—C13—C14—C9	−81.3 (8)	C45—C46—C47—C48	83.7 (8)
C12—C13—C14—C15	28.2 (6)	C45—C46—C49—C48	−81.6 (8)
C13—C12—C15—C14	28.0 (6)	C45—C46—C49—C50	39.5 (12)
C13—C12—C15—C16	148.5 (8)	C45—C46—C49—C51	166.8 (8)
C13—C12—C15—C17	−83.9 (8)	C46—C47—C48—C43	−81.3 (8)
C13—C14—C15—C12	−27.8 (6)	C46—C47—C48—C49	27.8 (6)
C13—C14—C15—C16	−148.3 (8)	C47—C46—C49—C48	27.4 (6)
C13—C14—C15—C17	84.3 (8)	C47—C46—C49—C50	148.5 (9)
C14—C9—C10—N2	−178.3 (8)	C47—C46—C49—C51	−84.2 (8)
C14—C9—C10—C11	0.5 (11)	C47—C48—C49—C46	−27.3 (6)
C15—C12—C13—C14	−28.2 (6)	C47—C48—C49—C50	−147.3 (8)
C18—N3—C22—C21	−0.1 (12)	C47—C48—C49—C51	84.3 (8)
C18—N3—C22—C23	178.7 (7)	C48—C43—C44—N6	179.6 (7)
C18—C19—C20—C21	−2.8 (13)	C48—C43—C44—C45	0.9 (11)
C19—C20—C21—C22	2.3 (13)	C49—C46—C47—C48	−27.7 (6)

### Hydrogen-bond geometry (Å, º)

**Table:**

*D*—H···*A*	*D*—H	H···*A*	*D*···*A*	*D*—H···*A*
O1—H1*A*···Cl1	0.84 (10)	2.37 (10)	3.194 (7)	166 (9)
O1—H1*B*···Cl4^i^	0.87 (10)	2.43 (10)	3.260 (7)	161 (9)

Symmetry code: (i) −*x*+1, *y*+1/2, −*z*+1/2.
